# Strength Enhancement of Clay Through Lime–Sand Stabilization at Various Remolding Water Contents

**DOI:** 10.3390/ma18143282

**Published:** 2025-07-11

**Authors:** Shuai Qi, Jinhui Liu, Wei Ma, Jing Wang

**Affiliations:** 1 School of Civil and Transportation Engineering, Beijing University of Civil Engineering and Architecture, Beijing 102616, China; 2108590023152@stu.bucea.edu.cn (J.L.); 2108590022164@stu.bucea.edu.cn (W.M.); 202002020429@stu.bucea.edu.cn (J.W.); 2 Beijing Urban Transportation Infrastructure Engineering Technology Research Center, Beijing 102616, China

**Keywords:** clay soils, lime–sand stabilized clay, enhancement effectiveness, remolding water content, unconfined compression strength

## Abstract

During the construction of subgrade, the remolding water content *w* of lime–sand-stabilized clay usually varies in a wide range, leading to inconsistent effectiveness in strength enhancement. Until now, this aspect has not been investigated. In this study, an unconfined compression test and microscopic observation were carried out on clay and stabilized clay (adding 4% lime by mass and 50% sand by volume). The results show the following: (1) remolding water content *w* had a strong effect on the soil fabrics of pure clay and lime-stabilized clay. An increase in the w from the dry to wet side of optimum reduced matric suction, which diminished the aggregation effect among fine-grained particles in both clay and lime-stabilized clay. Correspondingly, fine-grained aggregate progressively disintegrated, and dispersed fine-grained particles increased. As a result, the *w* increment at *w* ≤ *w*_cha_ made the dispersed fine-grained particles successively fill the large pores between aggregates, densifying the soil fabric. In contrast, at *w* > *w*_cha_, the ongoing disintegration of aggregate resulted in progressive structural weakening. Herein, *w*_cha_ was defined as the characteristic water content at which the soil fabric transitioned from structural densification to weakening. (2) The UCS of both pure clay and lime–sand-stabilized clay followed a bell-shaped pattern as the *w* increased, with *w*_cha_ acting as the turning point. For pure clay soils, the UCS increased with increasing *w* up to *w*_cha_ because of structural densification, but decreased beyond *w*_cha_ due to structural weakening. In lime–sand-stabilized clay, where a sand grain skeleton developed, the compression of lime-stabilized clay induced by the movement of sand grains during shearing activated its contribution to the overall strength. The compressive capacity of the lime-stabilized clay varied in a bell-shaped manner with *w*, and this trend was mirrored in the UCS of lime–sand-stabilized clay. (3) At a low *w*, the fact that the clay aggregate exhibited sand-like mechanical behavior reduced the effectiveness of incorporating sand and lime for enhancing the UCS. As the *w* increased at *w* ≤ *w*_cha_, the breakdown of aggregates enlarged the distinction between pure clay and sand, resulting in a more pronounced improvement in the UCS with the addition of sand and lime. At *w* > *w*_cha_, the lubrication effect occurring at the contact between sand grains diminished the interlocking between the sand grains. Consequently, the effectiveness of the UCS enhancement decreased.

## 1. Introduction

Subgrade is a critical structural component of road infrastructure, serving to support overlying pavement systems and distribute traffic loadings to the underlying ground. During the construction of subgrade, clay soils are usually utilized as filling materials, owing to their economic benefits and their strength property, playing an important role in ensuring the stable and safe operation of road infrastructure. Currently, with the continuous increase in demands for transportation efficiency and load-bearing capacity, subgrade is subjected to progressively higher levels of traffic loading stress. This has led to more stringent requirements for the strength property of subgrade filling materials. However, untreated clay soils typically exhibit low shear strength, which may cause severe engineering problems (such as excessive settlement) when directly employed as filling materials. In response to this challenge, many soil stabilization techniques have been introduced, among which the combined use of lime and sand has proven to be effective in improving the strength property of pure clay soils in engineering practice [1].

According to specification [2], lime–sand-stabilized clay soils should be compacted at optimum water content *w*_opt_. However, in field construction, the remolding water content *w* is difficult to control precisely due to complex in situ conditions and may vary across a wide range. This variability in the *w* may lead to fluctuations in the effectiveness of lime–sand stabilization in enhancing clay strength. Therefore, from a practical point of view, it is important to investigate how remolding water content *w* affects the effectiveness of lime–sand stabilization in terms of strength.

Until now, extensive investigations have been carried out focusing on the utilization of sand or lime as individual additives for improving the mechanical behavior of clay soils. These investigations were commonly carried out under a fixed *w* value. Numerous studies have shown that the incorporation of sand into fine-grained soils can significantly improve their engineering properties. Dong et al. [3] found that at optimum water content *w*_opt_ = 17.8%, the addition of sand to red clay with a high liquid limit could minimize volume expansion upon hydration and enhance mechanical strength. In a study by Liu et al. [4], it was observed that adding sands to red clay would increase its California Bearing Ratio (CBR) value, thereby meeting the requirements of subgrade materials in highway construction. Alnmr et al. [5] observed that incorporating more than 30% sand (by mass) into expansive soils results in a significant change in soil fabric, which effectively suppresses both compressibility and swelling properties. Dafalla et al. [6] reported that the contraction behavior of clay soils was reduced due to the inclusion of sand grains. Similarly to the effect of sands, lime stabilization at a fixed *w* value has been shown to significantly enhance the engineering properties of clay soils. Bessaim et al. [7] reported that lime addition reduced the plasticity index of clay soils from 21% to 15%, indicating a significant improvement in workability. Nejib Ghazouani et al. [8] demonstrated that the addition of 4% lime (by mass) significantly enhanced the stability and bearing capacity of tuff. Wang et al. [9,10,11] also reported that lime improved the stiffness of clay soils, as evidenced by the increase in shear modulus. Additionally, Shi et al. [12] reported that increasing the lime content led to higher cohesion, with little change in friction angle. In a study by Wang et al. [13] of clay soils, it was found that after being stabilized with lime, their durability under freeze–thaw cycles became enhanced. A similar phenomenon was observed in the study of Li et al. [14] with silty clay. When investigating lime-stabilized clay under thermal cycles, Tunono et al. [15] reported that lime stabilization enhanced the soil’s resistance. Note that lime stabilization can significantly alter the microstructure of fine-grained soils, often leading to shrinkage due to pore size redistribution and particle aggregation. Studies by Rosone et al. have shown that water consumption during stabilization increases matric suction, promoting internal contraction even under static conditions. This moisture-driven shrinkage may induce stress and cracking in subgrades, even in the absence of direct traffic loading.

Despite these extensive studies, most existing studies have focused on either sand or lime modification alone, and were primarily conducted under fixed water content condition. To date, only Yang et al. [16] have reported that at low water content, the incorporation of sand significantly inhibited the swelling behavior of expansive soils, and this inhibiting effect was reduced as the *w* increased. When the *w* exceeded 12%, the variation in swelling behavior became negligible. Muawia Dafalla et al. [17] found that adding 8% lime (by mass) to expansive soil at optimum water content *w*_opt_ would significantly enhance its unconfined compressive strength (UCS). But as the water content *w* exceeded *w*_opt_, the UCS would decrease. To the author’s knowledge, there is a lack of investigation into the strength behavior of clay soils stabilized with a combined application of lime and sand under varying remolding water contents. The effectiveness of this composite modification method across a wide range of *w*, particularly in terms of enhancing strength properties, has remained largely unexplored.

To address this gap, a systematic experimental study was designed to evaluate the effectiveness of combined lime–sand stabilization in improving the strength properties of clay soils under varying remolding water contents. Unconfined compression tests were carried out on untreated and lime–sand-stabilized clay soils under seven different remolding water contents (*w* = 14%, 16%, 20%, 25%, 28%, 30%, and 35%), covering both the optimum moisture content *w*_opt_ as well as on the dry and wet sides. In addition, microstructural observations were made on representative samples, aiming to elucidate the underlying mechanisms contributing to the observed mechanical behavior.

## 2. Materials and Methods

### 2.1. Testing Materials

**Figure 1:**
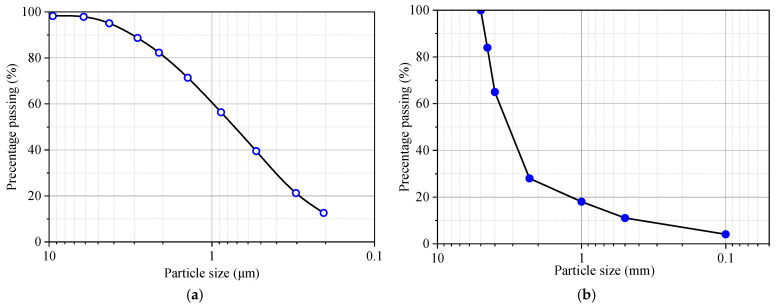
Grain size distribution curve: (**a**) clay soils; (**b**) sand grains.

**Figure 2:**
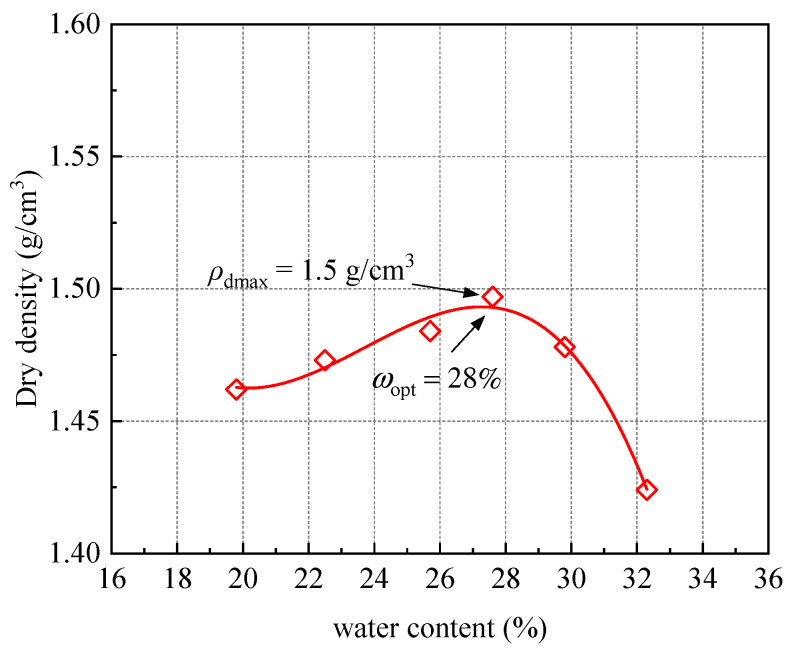
Compaction curve of tested clay soils.

The clay soil used for stabilization in this study was a typical clay filling encountered in subgrade construction (see its grain size distribution in Figure 1a). This clay filling had a liquid limit *w*_L_ = 63% and a plasticity index *I*_p_ = 26%. According to the Unified Soil Classification System (USCS) detailed by ASTM D422-63 [18], it is classified as CH. The chemical composition of the clay soil was determined through X-ray fluorescence (XRF) (see the results in Table 1). The results indicated that SiO_2_ accounted for 47% by mass and Al_2_O_3_ accounted for 38%, with the remaining constituents classified as minor impurities. A standard compaction test was carried out to determine the compaction characteristic of the clay soil. The test yielded an optimum water content *w*_opt_ of 28% and a maximum dry density *ρ*_dmax_ of 1.50 g/cm^3^. The resulting compaction curve is presented in Figure 2.

To enhance the strength of the clay, a combined stabilization approach using lime and sand was applied. For the sand grains, a typical quartz sand was used, with its grain size distribution presented in Figure 1b. The sands had a median grain size of 4.95 mm and a uniformity coefficient *C*_u_ = 1.72 (summarized in Table 2). Regarding lime, a quicklime commonly used in subgrade construction was employed. The chemical composition of the quicklime is provided in Table 1. As can be seen, the content of CaO exceeded 97.3%, indicating a high content of active ingredients.

### 2.2. Experimental Methods

#### 2.2.1. Sample Preparation

This study used a cylindrical sample with a diameter *D* of 39.1 mm and a height *H* of 80 mm. The dimension of the sample was compatible with the equipment used for the unconfined compression test. The maximum grain size contained in a sample was 5 mm. The ratio of the sample diameter to the maximum grain size exceeded 5, suggesting that the grain size effect had a negligible impact on the testing results (ASTM D2850) [19].

The tested samples used in this study can be divided into two kinds: pure clay soils and clay stabilized with 4% lime (by mass) and 50% sand (by volume). Note that the sand content in the sample was evaluated in terms of volume rather than mass because volume provides a more accurate representation of the sand’s contribution to the formation of the sand skeleton. These additive ratios were selected based on commonly used values in the field. The fine-grained matrix in each group was compacted to achieve two target compaction degrees, *D*_c_ = 0.85 and 0.90 (corresponding to dry densities *ρ*_d_ of 1.26 g/cm^3^ and 1.34 g/cm^3^), which were consistent with the in situ conditions reported by Liu et al. [20]. Seven remolding water contents (*w* = 14%, 16%, 20%, 25%, 28%, 30%, and 35%) were selected for the fine-grained soils (clay and lime-stabilized clay) to cover the optimum water content *w*_opt_ = 28%, as well as on the dry and wet sides.

The sample was prepared through the following procedure. Firstly, the oven-dried lime was dry-mixed with the oven-dried clay using an electric mixer. This dry-mixing approach was adopted to promote uniform dispersion and effective blending of the lime and clay particles prior to the addition of water. Then, water corresponding to the target remolding water content was uniformly sprayed onto the blended fine-grained soils. The moistened soils were subsequently sealed in an airtight container and equilibrated for 24 h. Prior to compaction, the equilibrated fine-grained soils were either re-mixed thoroughly or blended with sand grains. Compaction was performed in five layers using a static compaction method. To guarantee the integration between adjacent layers, the top surface of each layer was scarified before the placement of the soils of the subsequent layer. After the completion of compaction, each sample was carefully demolded.

Following this procedure, all samples were carefully covered with a plastic membrane, placed into a hermetic box, and subjected to a curing period of 24 h in a temperature-controlled chamber maintained at 20 ± 2 °C. The selection of a 24 h curing period was primarily motivated by the need to simulate field conditions where rapid stabilization is essential, such as in emergency road construction or fast-track infrastructure projects. In these scenarios, early strength development is a critical design consideration. The UCS increment during this short period can be primarily attributed to two mechanisms: (1) the increase in matric suction due to water consumption during lime hydration, and (2) the flocculation of clay particles, which promote the formation of edge-to-surface contacts (Prusinski and Bhattacharja, 1999) [21].

#### 2.2.2. Unconfined Compression Test

A strain-controlled unconfined compression apparatus was used for the unconfined compression test. Prior to testing, careful alignment of the lower and upper plates was conducted with a vernier caliper to ensure uniform stress distribution in the sample during loading. During the test, the sample was placed at the center of the lower plate, which was then raised until the sample was fully in contact with the upper plate. Afterwards, strain-controlled compression was applied at a rate of 1 mm/min [22] until the sample failed. Throughout the loading, axial load and displacement were continuously recorded.

#### 2.2.3. Microscopic Tests

The microscopic tests included the mercury intrusion porosimetry (MIP) test and Micro-CT (μCT) test. It should be emphasized that the presence of moisture can significantly affect the reliability of µCT and MIP results. To mitigate this interference, each tested sample was subjected to a freeze-drying process before testing. This process effectively removed water through rapid freezing with liquid nitrogen, followed by vacuum sublimation drying, while preserving the original microstructure of the soil.

MIP tests were carried out using an AutoPore IV 9500 porosimeter (Micrometritics Corporate, Norcross, GA, USA) to investigate the pore size distribution in both pure clay and lime-stabilized clay. For the clay soils, three typical remolding water contents were selected, namely *w*_opt_ = 28%, a dry side (*w* = 20%), and a wet side (*w* = 35%), while for the lime-stabilized clay, the remolding water contents on the dry side (*w* = 20%) and wet side (*w* = 35%) were considered. In the mercury intrusion test, pressure was gradually increased to progressively drive mercury into soil pores, starting from large ones at low pressure to small ones at high pressure. For each pressure step, both the volume of intruded mercury and the corresponding pore diameter (calculated according to Delage et al. [23]) were obtained. μCT testing was conducted using a μCT scanner to observe the sand grain distribution. The scanning parameters included an accelerating voltage of 150 kV and a tube current of 150 μA, with a minimum pixel resolution of 44 μm. The μCT tests were performed on a sample with optimum water *w*_opt_ = 28%.

## 3. Results

### 3.1. Mechanical Behavior of Clay Soils

**Figure 3:**
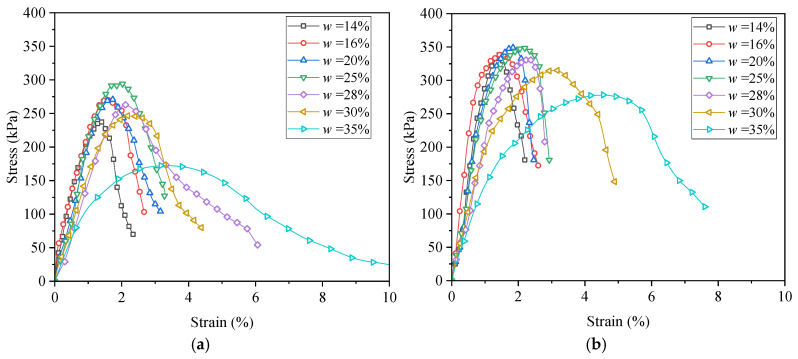
Stress–strain curves of clay at various remolding water content *w* values under two compaction degree *D*_c_ levels: (**a**) *D*_c_ = 0.85; (**b**) *D*_c_ = 0.90.

Figure 3 illustrates the stress–strain responses of the clay soils at different remolding water contents under two compaction levels (*D*_c_ = 0.85 and 0.90). A strain-softening pattern was identified for each stress–strain relationship, characterized by an initial increase in axial stress *q* with increasing axial strain *ε*_1_, reaching the unconfined compression strength UCS, followed by a gradual decline in *q* during further deformation.

**Figure 4:**
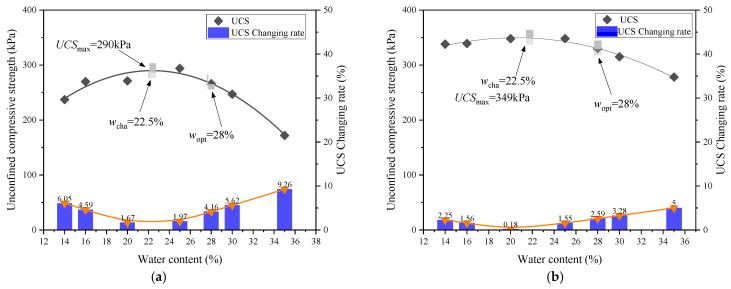
Variations in unconfined compression strength (UCS) and UCS changing rate with remolding water content *w* for clay soils under two compaction degree *D*_c_ levels: (**a**) *D*_c_ = 0.85; (**b**) *D*_c_ = 0.90.

As can be seen from Figure 3, the remolding water content *w* had a significant effect on the UCS. Overall, the relationship between the UCS and *w* was non-monotonic. To further investigate this *w* effect, Figure 4 depicts the variation in the UCS with *w* under two compaction degrees. It was obvious that at each *D*_c_ level, the UCS first increased and then decreased with increasing *w*, exhibiting a “bell-shaped” trend. In the study of Zhang et al. [24] focusing on the stiffness of silty clay with various *w* values, the variation in stiffness exhibited the same trend. At the peak point of UCS, a characteristic water content *w*_cha_ can be defined, which separates the *w* effect into two distinct regions: when the *w* ≤ *w*_cha_, the *w* increment enhanced the UCS, while when the *w* > *w*_cha_, the UCS decreased with a further increase in *w*. Moreover, the *w*_cha_ values were identical for two *D*_c_ levels (*w*_cha_ = 22.5%), and this value was lower than the optimum water content, *w*_opt_ = 28%.

**Figure 5:**
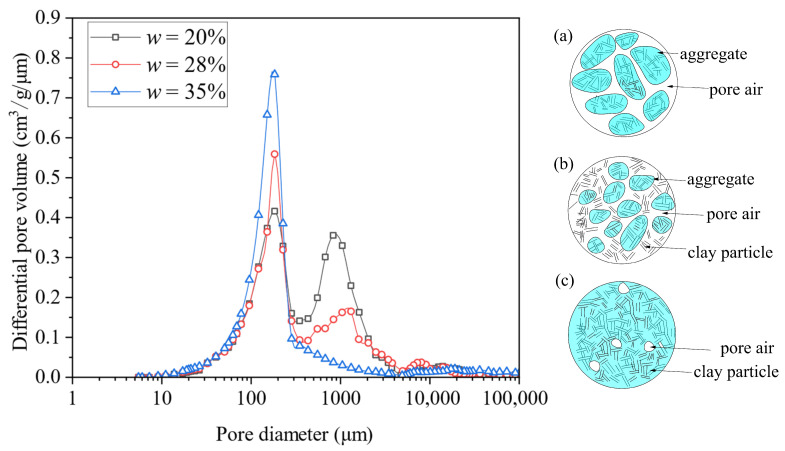
Pore size distributions of clay soils with typical *w* values and the corresponding soil fabric: (**a**) aggregate–aggregate structure (*w* = 20%); (**b**) aggregate-dispersed structure (*w* = 28%); (**c**) dispersed structure (*w* = 35%).

This variation in the UCS with *w* was closely related to the microstructure evolution of the compacted clay soils. The pore size distributions at *w*_opt_ = 28%, as well as on the dry side (*w* = 20%) and wet side (*w* = 35%), obtained from the MIP tests are depicted in Figure 5. It can be observed that at *w*_opt_ and on the dry side, the pore distribution exhibited a bimodal distribution, with a boundary at pore diameter *d* = 300 μm, consisting of a large-pore group (*d* > 300 μm) and a small-pore group (*d* ≤ 300 μm). In addition, compared with the case on the dry side, there existed more small pores and fewer large pores at *w*_opt_. When the water content *w* was on the wet side, the pore structure displayed a unimodal distribution characteristic, dominated by small pores (*d* ≤ 300 μm). Similar observations were made by Li and Zhang [25] and Delage [23].

This pore structure evolution reflected the relative proportion of dispersed clay particle and clay aggregate (Figure 5a–c). When on the dry side, the high matrix suction exerted a strong aggregation effect between clay particles [26], forming an aggregate–aggregate structure (Figure 5a). At *w*_opt_ = 28%, the reduced suction weakened the aggregation effect, resulting in smaller aggregates and more dispersed particles, thus forming an aggregate-dispersed structure (Figure 5b). When *w* exceeded *w*_opt_, the aggregation effect nearly disappeared since the matrix reached a pretty low level, leading to a fully dispersed structure (Figure 5c).

When clay was on the dry side, numerous large pores between aggregates resulted in a loose structure and low UCS value. As *w* increased, the dispersed clay particles gradually filled these large pores, densifying the soil structure. At *w*_cha_ = 22.5%, the large pores were well filled, resulting in the highest structural stability and peak UCS (UCS_max_). Beyond *w*_cha_ = 22.5%, the aggregates gradually diminished and eventually disappeared, while the reduced matric suction weakened the bonding effect on the adjacent clay particles. Consequently, the soil structure became progressively weaker and the UCS showed a decreasing trend.

### 3.2. Mechanical Behavior of Lime–Sand-Stabilized Clay

**Figure 6:**
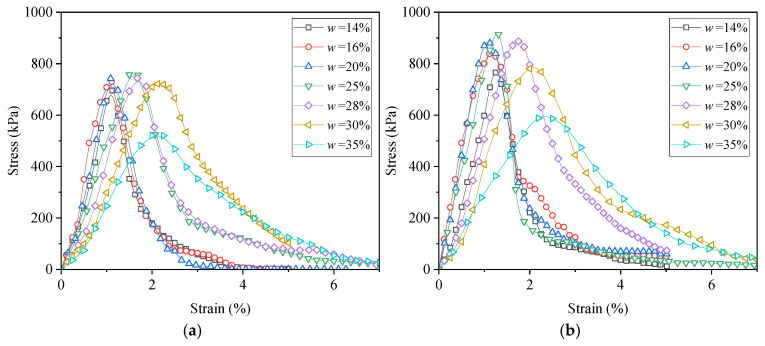
Stress–strain curves for lime–sand-stabilized clay at various *w* values under two compaction degree *D*_c_ levels: (**a**) *D*_c_ = 0.85; (**b**) *D*_c_ = 0.90.

**Figure 7:**
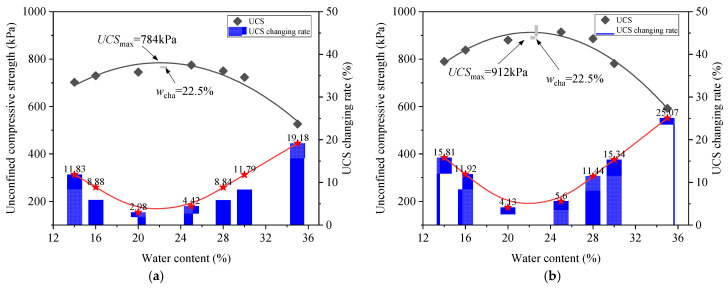
Variations in unconfined compression strength (UCS) and UCS changing rate with *w* for lime–sand-stabilized clay at two compaction degree *D*_c_ levels: (**a**) *D*_c_ = 0.85; (**b**) *D*_c_ = 0.90.

The stress–strain curves of lime–sand-stabilized clay at various remolding water contents are plotted in Figure 6. Similarly to the clay sample, each stabilized clay sample exhibited strain-softening behavior, and it seemed that the UCS varied non-monotonically with increasing *w*. The UCS-*w* relationship curve depicted in Figure 7 displays the *w* effect on the UCS more clearly. It can be observed that the UCS of the stabilized clay also exhibited a typical “bell-shaped” pattern in response to variation in *w*. At the point of peak UCS (UCS_max_), a characteristic water content *w*_cha_ can be identified, separating the *w* effect into two distinct zones: the UCS increased when *w* ≤ *w*_cha_, but decreased when *w* > *w*_cha_. Notably, the characteristic water content of *w*_cha_ = 22.5% of the lime–sand-stabilized clay is identical to that of the pure clay, which remained below the *w*_opt_ = 28%. This indicates that compaction can be effectively carried out on the dry side of *w*_opt_. Indeed, compaction at lower water contents can sometimes lead to volumetric problems. Future studies should consider these volumetric behaviors to better assess the field applicability of lime–sand stabilization and ensure long-term performance under field conditions.

**Figure 8:**
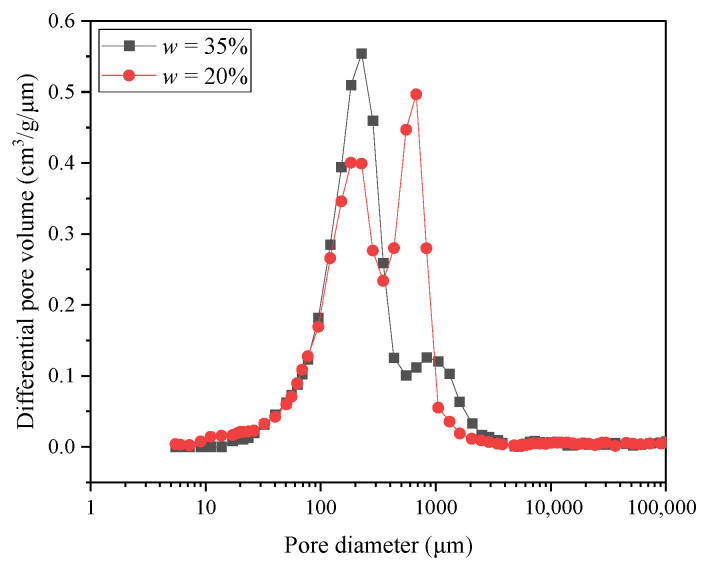
Pore size distributions of lime-stabilized clay at *D*_c_ = 0.85 under two remolding water content *w* levels.

This UCS variation with *w* for sand–lime-stabilized clay was highly related to the soil fabric evolution. The entire sample can be regarded to be consist of two parts: sand grains and the lime-stabilized clay. For the lime-stabilized clay part, the MIP results (see Figure 8) show that the pore size distribution exhibited a bimodal characteristic on the dry side (*w* = 20%), while on the wet side (*w* = 35%), a unimodal distribution was observed. This variation is consistent with that of pure clay, indicating that the addition of lime did not alter the fundamental pattern of pore size distribution. The pore structure variation in lime-stabilized clay also illustrates the varying ratio between the dispersed fine-grained particle and the fine-grained aggregate, which was the same as the case of pure clay. Under dry conditions, an aggregate–aggregate structure was formed due to the aggregate formation induced by the high matric suction. When the remolding water content increased, the diminishing matric suction weakened the aggregation process, leading to the formation of more dispersed fine-grained particles. On the wet side, the aggregation process almost disappeared due to the negligible matric suction, and the fabric of the fine-grained soil became dominated by dispersed fine-grained particles.

Consistent with this varying ratio between the dispersed fine-grained particle and fine-grained aggregate, the UCS variation in the lime-mixed clay also exhibited a “bell-shaped” characteristic (see Figure 7). At a low water content, large pores formed between aggregates, leading to a loose structure and small UCS value. As the *w* rose, increased quantities of dispersed particles gradually filled the large pores between aggregates, leading to fabric densification. At *w*_cha_, pore filling reached the optimal state, which yielded the maximum UCS (UCS_max_). Beyond this, aggregates successively disappeared and the reduced matric suction weakened the bonding, causing the UCS to decline.

**Figure 9:**
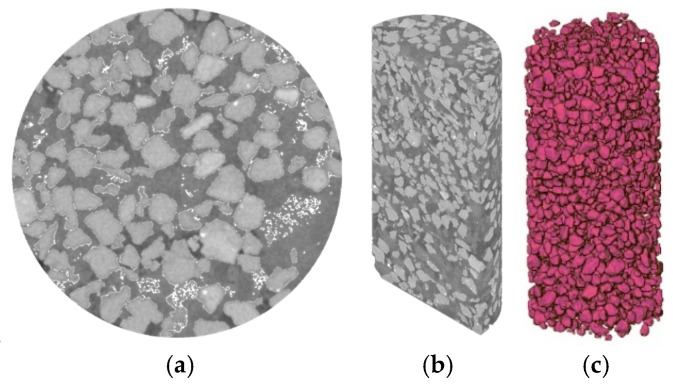
μCT scanning results in as-compacted sample: (**a**) cross-section view; (**b**) vertical section view; (**c**) three-dimensional view of sand grain distribution.

Regarding the sand grain, its three-dimensional distribution inside the sample (μCT scanning results) shows that the sand grains were inter-connected with each other, with a sand grain skeleton formed (see Figure 9). Indeed, following the method proposed by Monkul and Ozden 2007 [27], in the sand used in this study, when volumetric sand content reached 49.2%, a sand grain skeleton started to be formed. In this case, most of the fine-grained matrix (namely, lime-stabilized clay) was located within the large pores formed by the sand skeleton. Under shear loading, the movement of the sand grains exerted compressive stress on the lime-mixed clay within these pores, thereby activating its contribution to the overall strength of the entire sand–lime stabilized clay. As mentioned above, as the remolding water content *w* of the fine-grained matrix (lime-stabilized clay) increased from the dry side to *w*_cha_, the amount of dispersed fine-grained particles gradually increased and filled the pores between the aggregates, leading to the densification of the fine-grained matrix structure. This process enhanced the compressive stiffness of the fine-grained matrix within the macropores between sand grains, resulting in a continuous increase in the UCS with rising *w*. When the *w* exceeded *w*_cha_ and continued to increase, the progressive disintegration of fine-grained aggregates further increased the proportion of dispersed particles, which weakened the compressive stiffness of the fine-grained matrix. Consequently, the UCS began to decrease.

### 3.3. Comparison of Mechanical Behavior Between Clay and Lime–Sand-Stabilized Clay

**Figure 10:**
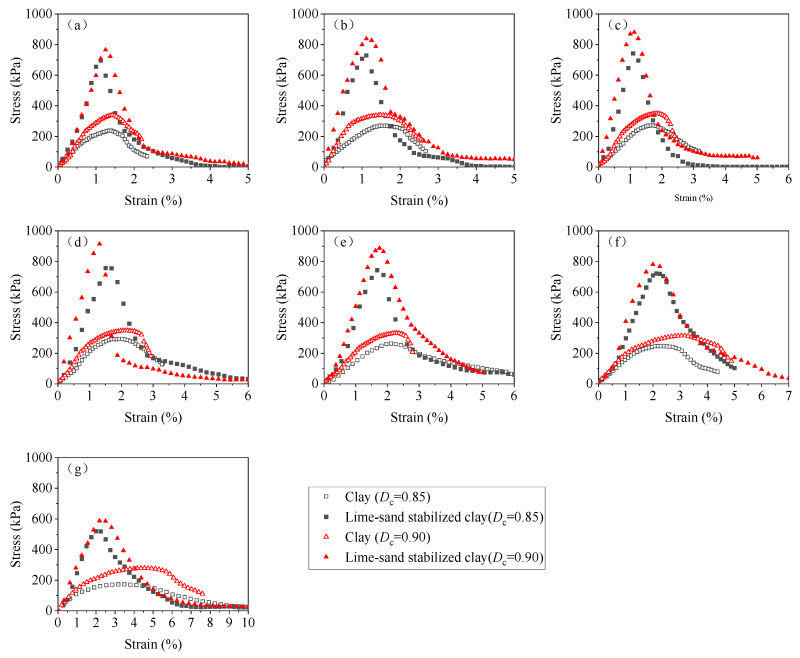
Comparison of stress–strain curve between clay and lime–sand-stabilized clay at various *w* values: (**a**) *w* = 14%; (**b**) *w* = 16%; (**c**) *w* = 20%; (**d**) *w* = 25%; (**e**) *w* = 28%; (**f**) *w* = 30%; (**g**) *w* = 35%.

Figure 10 presents a comparison of the stress–strain relationship between clay and lime–sand-stabilized clay. As can be clearly observed, at each remolding water content *w*, the addition of lime and sand led to a significant improvement in the UCS of the clay.

**Figure 11:**
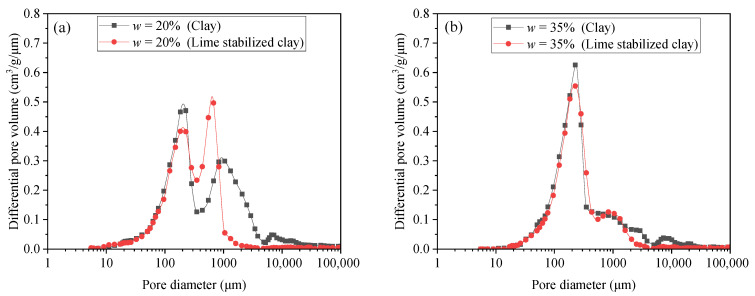
Comparison of pore size distribution between clay and lime-stabilized clay at compaction degree *D*_c_ = 0.85 under two remolding water content *w* levels: (**a**) *w* = 20%; (**b**) *w* = 35%.

This phenomenon was strongly supported by the μCT results and the MIP results. As shown in the μCT results (Figure 9), the addition of sand grains caused the formation of a sand grain skeleton with interlocking contacts between adjacent grains, which significantly improved the frictional resistance relative to the pure clay. In the MIP results, when on the dry side (*w* = 20%) (see Figure 11a), adding lime shifted the modal pore diameter of the large-pore group (*d* > 300 μm) from 936 μm to 653 μm, while the total volume of large pores remained relatively unchanged (0.19 cm^3^/g before liming and 0.18 cm^3^/g after liming). When on the wet side (*w* = 35%), lime addition resulted in an overall reduction in the pores (see Figure 11b). It is worth noting that given the short curing time (24 h), this observed pore structure variation was predominantly driven by flocculation and agglomeration within the clay particles, which led to the formation of edge–surface contacts among them (Prusinski and Bhattacharja, 1999) [21]. Under the combined effects of these structural changes, the UCS exhibited an increasing trend. It is important to note that among the two stabilizing agents (sand and lime), the improvement in the UCS is primarily attributed to the addition of sand. The substantial quantity of sand added fundamentally altered the soil structure, transitioning it from a clay-dominated matrix to a sand skeleton structure. In contrast, lime played a secondary role, mainly influencing the soil fabric through its effects on clay particle flocculation and aggregation.

**Figure 12:**
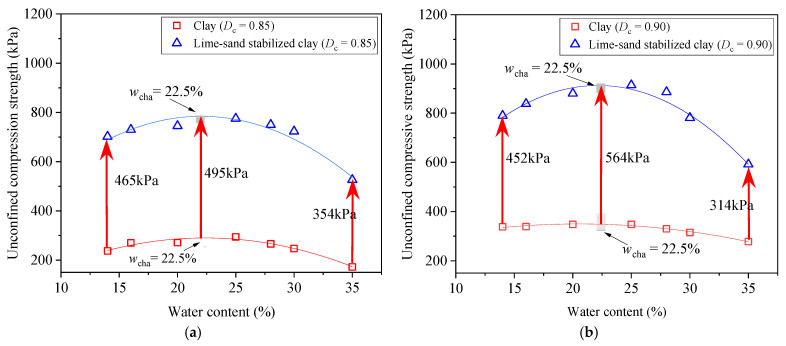
Comparison of UCS-*w* relationship between clay and lime–sand-stabilized clay at two compaction degree *D*_c_ levels: (**a**) *D*_c_ = 0.85; (**b**) *D*_c_ = 0.90.

To better understand the effect of the addition of lime and sand on the UCS under different remolding water contents *w*, Figure 12 presents a comparison of the UCS-*w* relationships between the pure clay and the lime–sand-stabilized clay. These relationships are described using a quadratic function. The coefficient of determination (*R*^2^) values were calculated for both clay and lime–sand-stabilized clay at two compaction degrees (*D*_c_ = 0.85 and *D*_c_ = 0.90). Specifically, the *R*^2^ values were 0.96 and 0.98 for clay, and 0.93 and 0.96 for lime–sand-stabilized clay at *D*_c_ = 0.85 and *D*_c_ = 0.90, respectively. These high *R*^2^ values indicate that the quadratic model fitted the experimental data well.

It can be observed that at each compaction degree *D*_c_, when at *w* = 14%, the increment in the UCS (ΔUCS) exhibited a relatively small level: ΔUCS = 465 kPa at *D*_c_ = 0.85, ΔUCS = 452 kPa at *D*_c_ = 0.90. When the *w* increased within the rage *w* ≤ *w*_cha_, ΔUCS gradually increased, reaching the maximum level at *w*_cha_ = 22.5% (ΔUCS = 495 kPa at *D*_c_ = 0.85, ΔUCS = 564 kPa at *D*_c_ = 0.90). Beyond *w*_cha_, ΔUCS started to decrease and at *w* = 35%, ΔUCS reduced to 354 kPa at *D*_c_ = 0.85 and 314 kPa at *D*_c_ = 0.90. This variation in UCS enhancement with varying *w* can be explained as follows.

At *w* = 14%, the soil fabric was dominated by fine-grained soil aggregate. Due to the pretty high matric suction within the aggregate, the aggregate possessed high resistance to breakdown under external loading. Compared to the dispersed fine-grained particles, these aggregates were more similar to sand grains [28]. Consequently, the addition of sand and lime had minimal effect on the overall soil fabric, resulting in the smallest improvement in the UCS (ΔUCS).

As the *w* gradually increased towards *w*_cha_, the quantity of clay aggregates decreased while the proportion of dispersed fine-grained particles increased. This shift enhanced the contrast between the pure clay and the added sand grains, thereby amplifying the enhancement effect of the addition of sand and lime on soil properties. As a result, the UCS enhancement (ΔUCS) became progressively more significant.

When *w* exceeded *w*_cha_ and continued to increase, the number of aggregates further declined, and the amount of dispersed particles kept rising. Meanwhile, the increased presence of fine particles at the contact interfaces between the sand grains enhanced the lubrication effects, which weakened the inter-grain interlocking. Therefore, in this *w* range, the UCS enhancement (ΔUCS) began to decline slightly with *w* increasing.

Note that the maximum UCS enhancement was achieved at a remolding water content of *w*_cha_ = 22.5%, which lies on the dry side of the optimum water content (*w*_opt_ = 28%). Although lime–sand stabilization proved most effective in improving clay strength under a low water level, field compaction under such conditions typically required greater effort due to the enhanced particle friction and bonding, potentially leading to higher energy consumption and construction costs. Therefore, in practical applications, it is essential to consider a balance between achieving favorable mechanical performance and maintaining compaction efficiency.

### 3.4. Comparison with Previous Studies

**Figure 13:**
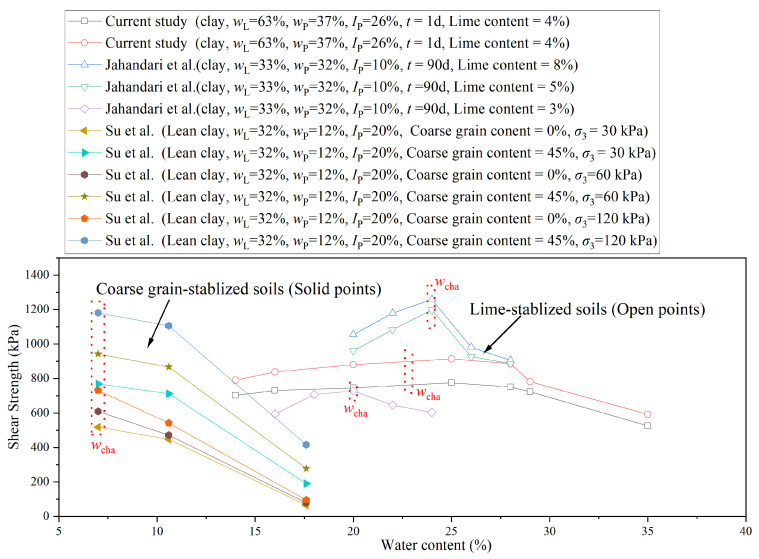
Comparison of testing data between current study and previous studies [29,30].

The relevant data from the literature were extracted and are plotted in Figure 13. Su et al. [29] investigated the effect of incorporating coarse grains into fine-grained soils and observed that the strength of the stabilized clay generally decreased with increasing water content, although an initial increase may occur at low water levels. This led to the definition of a characteristic water content *w*_cha_, which delineates the two distinct behavioral regimes. Jahandari et al. [30] reported a clear transition in strength development when lime was used for soil stabilization. Their findings also support the existence of a characteristic water content *w*_cha_ that separates the mechanical response into two distinct stages. In our study on lime–sand stabilization, we identified a comparable characteristic water content *w*_cha_, which effectively divided the strength behavior into two phases: an initial strengthening phase at lower water contents and a weakening phase beyond *w*_cha_. This observation is highly consistent with the previous studies cited above, which used lime or sand as an individual stabilizing agent.

## 4. Conclusions

This study carried out unconfined compression tests on clay and lime–sand-stabilized clay (adding 4% lime by mass and 50% sand by volume) at seven remolding water contents (*w* = 14%, 16%, 20%, 25%, 28%, 30%, and 35%). In addition, typical samples were selected for μCT and MIP observations. Based on the obtained results, the effectiveness of combined lime–sand stabilization in improving the strength properties of clay soils under varying remolding water contents could be evaluated, and the following conclusions were drawn.

(1) The soil fabrics of both pure clay and lime-mixed clay were significantly influenced by the remolding water content *w*. As the *w* increased from the dry side of optimum to the wet side, the reduction in matric suction led to a weakening of the aggregation effect among fine-grained particles, resulting in progressive disintegration of fine-grained aggregate and an increase in dispersed fine-grained particles. Consequently, when the *w* increased to *w* ≤ *w*_cha_, the dispersed fine-grained particles gradually filled the large pores between aggregates, leading to continuous densification of the soil fabric. In contrast, at *w* > *w*_cha_, the ongoing disintegration of the aggregate resulted in progressive structural weakening.
(2) As the *w* increased, both the pure clay and lime–sand-stabilized clay exhibited a bell-shaped UCS variation with *w*_cha_ as the threshold. In pure clay soils, the increase in the UCS with increasing *w* at *w* ≤ *w*_cha_ was due to structural densification, while the UCS decrement at *w* > *w*_cha_ resulted from structural weakening. For lime–sand-stabilized clay in which a sand grain skeleton had formed, the shearing-induced compression of lime-mixed clay by the sand grains activated its contribution to the overall strength. As the compressive capacity of the lime-mixed clay exhibited a bell-shaped response to *w*, with *w*_cha_ serving as the threshold, the overall UCS of the lime–sand-stabilized clay followed a similar pattern.
(3) At a low *w* value, the dominance of the clay aggregate with sand-like mechanical behavior limited the effectiveness of adding sand and lime in improving the UCS. As the *w* approached *w*_cha_, the disintegration of the aggregate enlarged the contrast between pure clay and sand, leading to a greater improvement in the UCS. Beyond *w*_cha_, the lubrication effect in sand–sand contact areas reduced the grain interlocking effect, slightly decreasing the effectiveness of UCS enhancement.

The above findings indicate that the maximum UCS enhancement was achieved at a remolding water content of *w*_cha_ = 22.5%. Both higher and lower water contents resulted in a reduction in the effectiveness of lime–sand stabilization. Therefore, in engineering practice, it is recommended to control the remolding water content around *w*_cha_. However, it should be noted that this *w*_cha_ value lies on the dry side of the optimum water content (*w*_opt_ = 28%). Compaction under such low water levels generally demands more effort due to increased interparticle resistance, which may result in higher energy consumption and elevated construction costs. Hence, a balance between achieving favorable mechanical performance and maintaining compaction efficiency should be considered in practical application.

It is important to note that this study was focused on a single type of clay with specific plasticity characteristics. Therefore, the findings may not be directly applicable to other fines-grained soils with different plasticity properties. Further investigations involving soils with other plasticity indexes are recommended.

## Figures and Tables

**Table 1:** Chemical composition of clay and lime.

Chemical Composition	Quicklime	Clay
CaO	>97.3%	None
SiO_2_	None	47%
Al_2_O_3_	None	38%

**Table 2:** Soil properties.

Soil Type	Index Property	Value
Clay soils	Specific gravity, *G*_s_	2.60
	Clay content (<2 μm)	85%
	Liquid limit, *w*_L_	63%
	Plastic limit, *w*_P_	37%
	Plasticity index, *I*_p_	26%
	USCS classification	CH
	Optimum water content, *w*_opt_	28%
	Maximum dry density, *ρ*_dmax_	1.50
Sand	Mean grain size	4.95 mm
	Coefficient of uniformity, *C*_u_	1.72
	Water absorption	0.3%
	Specific gravity, *G*_s_	2.66
	Plasticity index, *I*_p_	Non-plastic

## Data Availability

The original contributions presented in this study are included in the article. Further inquiries can be directed to the corresponding author.

## Abbreviations

The following abbreviations are used in this manuscript:

*C*_u_	coefficient of uniformity
*d*	pore diameter
*D*	sample diameter
*D_c_*	compaction degree
*G*_s_	specific gravity
*H*	sample height
*I*_p_	plasticity index
*q*	axial stress
UCS	unconfined compression strength
UCS_max_	maximum value of unconfined compression strength
*w*	remolding water content
*w*_cha_	characteristic water content
*w*_L_	liquid limit
*w_opt_*	optimum water content
*w*_p_	plastic limit
*ρ*_d_	dry density
*ρ*_dmax_	maximum dry density
*ε*_1_	axial strain
ΔUCS	variation value of UCS
